# Congenital Insensitivity to Pain and Anhidrosis With Orthopedic and Self‐Injury Complications in a 5‐Year‐Old Boy: A Case Report

**DOI:** 10.1002/ccr3.70004

**Published:** 2024-12-15

**Authors:** Zubair Amin, Humza Saeed, Syed Imam Naufil, Sadaf Saba, Abdullah Imtiaz, Syed Hassan Akhlaq, Muhammad Husnain Ahmad, Masab Ali

**Affiliations:** ^1^ Department of Internal Medicine Rawalpindi Medical University Rawalpindi Pakistan; ^2^ Department of Orthopedics Benazir Bhutto Hospital Rawalpindi Pakistan; ^3^ Department of Internal Medicine Pakistan Ordinance Factory Hospital Wah Cantt Pakistan; ^4^ Department of Internal Medicine Fatima Memorial Hospital College of Medicine and Dentistry Lahore Pakistan; ^5^ Tentishev Satkynbai Memorial Asian Medical Institute Kant Kyrgyzstan; ^6^ Department of Internal Medicine Punjab Medical College Faisalabad Pakistan

**Keywords:** congenital insensitivity to pain and anhidrosis, orthopedic fractures, recurrent fevers, self‐mutilation

## Abstract

Congenital insensitivity to pain with anhidrosis (CIPA) is a rare autosomal recessive disorder because of NTRK1 gene mutations, leading to an inability to perceive pain and temperature and lack of sweating. Its rarity and unique clinical challenges, such as severe injuries from the inability to sense pain, make reporting cases critical. A 5‐year‐old boy, the third child of consanguineous parents, was referred for a fractured femur. His history includes recurrent fevers, pain insensitivity, self‐mutilation, and anhidrosis with compensatory hyperhidrosis. Examination showed multiple ulcers, dry skin, missing digits, dental issues, and corneal ulcers. The neurological assessment confirmed loss of pain and temperature sensation. Genetic testing confirmed NTRK1 mutations, diagnosing CIPA. The femur fracture was treated with a hip spica cast, and injury prevention and temperature management were advised to the parents. This case underscores the importance of early diagnosis and comprehensive management of CIPA, highlights the need for genetic counseling for at‐risk families, and provides insights into managing the condition's complex challenges. A multidisciplinary approach is essential to improve patient outcomes and quality of life.

**Summary**

- Congenital insensitivity to pain with anhidrosis (CIPA) presents unique challenges, including severe injuries and temperature dysregulation.
- Early diagnosis, genetic counseling, and a multidisciplinary approach are essential for injury prevention, comprehensive management, and improving the quality of life in affected patients, especially in consanguineous families.

## 1 Introduction

Congenital insensitivity to pain with anhidrosis (CIPA), also known as hereditary sensory and autonomic neuropathy type IV (HSAN IV), is a rare autosomal recessive disorder first described by Swanson in 1963 [1]. Characterized by an inability to perceive pain and temperature, along with a lack of sweating (anhidrosis), CIPA is caused by mutations in the NTRK1 gene. This gene is essential for the development and maintenance of sensory and autonomic neurons. Patients with CIPA exhibit a complete absence of unmyelinated fibers and a reduced number of small, myelinated fibers, leading to loss of pain, temperature sensation, and autonomic dysfunction [1, 2].

Individuals with CIPA often experience recurrent fevers, self‐mutilation, intellectual disability, and orthopedic issues because of their inability to feel pain. These symptoms frequently result in severe injuries, infections, and poor wound healing, thereby increasing the risks of morbidity and mortality. Early recognition and management are crucial, as there is currently no cure for CIPA. Prenatal screening and genetic counseling are important preventive measures for families with a history of CIPA [2, 3].

We present the case of a 5‐year‐old boy with CIPA who was presented with a fractured femur and other characteristic symptoms. This case aims to enhance the understanding of CIPA and provide health‐care professionals with insights into the importance of early recognition and multidisciplinary management of this condition.

## 2 Case Presentation

A 5‐year‐old male, resident of Kahuta, District Rawalpindi, presented to Benazir Bhutto Hospital in Rawalpindi, Pakistan, with a fractured femur sustained from a fall. The case raised significant concerns because of the presence of self‐inflicted injuries, burn marks, fractures, and ulcerations, suggesting an insensitivity to pain—a characteristic symptom of CIPA. He is the third child of consanguineous parents, and his two older siblings are reported to be healthy. The child's medical history is notable for recurrent fevers and injurious behaviors that began in infancy. His parents reported frequent engagement in harmful activities, such as biting his nails and striking hard or hot objects, resulting in severe damage to his fingers and toes. As a consequence, he has lost three toes and one thumb. Additionally, he presents with anhidrosis associated with episodes of hyperpyrexia and patches of compensatory hyperhidrosis.

**FIGURE 1:**
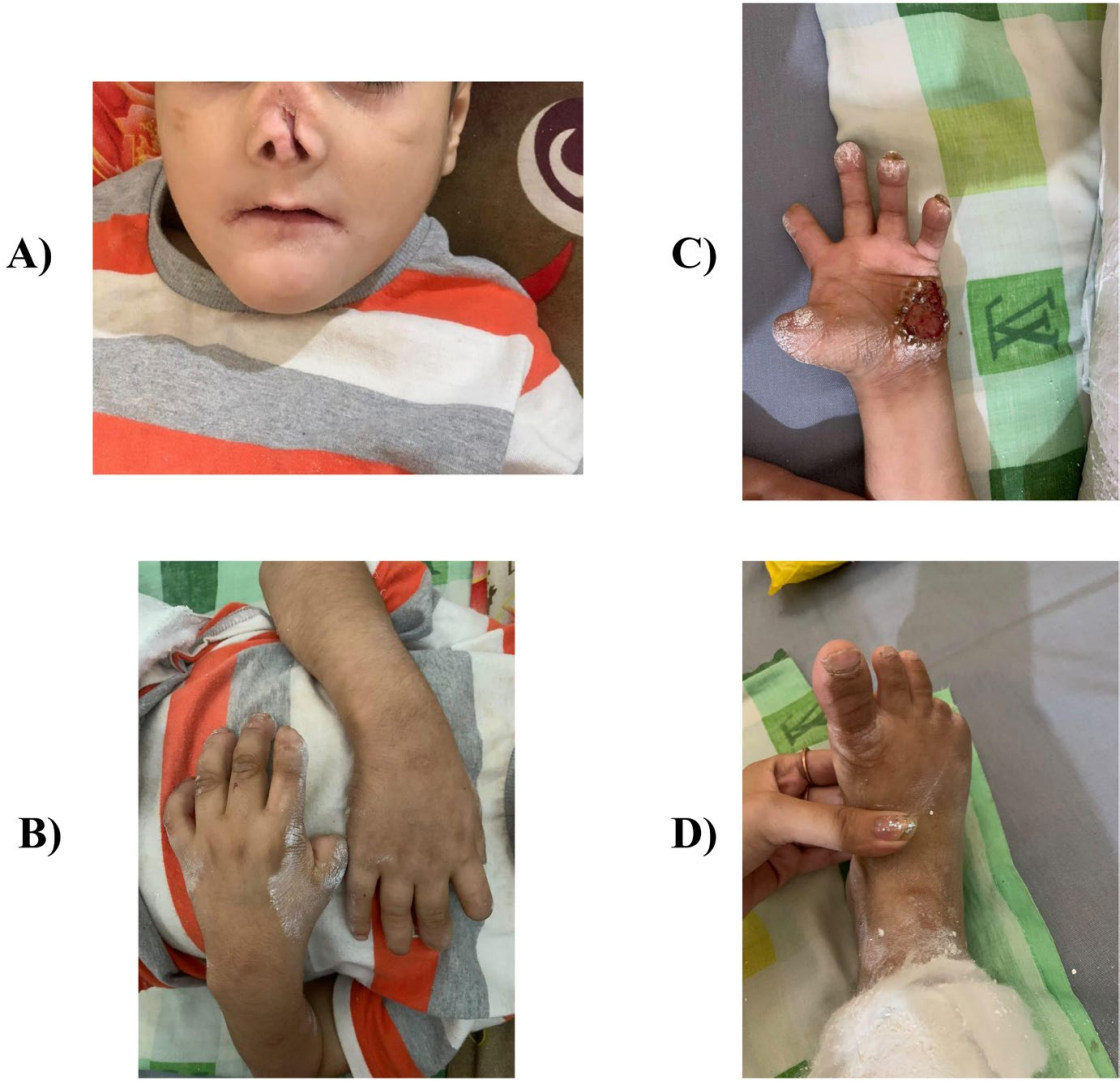
(A–D): Physical appearance of the child showing the absence of teeth, nasal damage, ulcers and damage to fingers and toes due to self‐mutilation.

On examination, the child displayed multiple ulcers and dry skin lesions, particularly around the oral cavity, eyes, palms, and soles, along with wound marks from self‐inflicted injuries (Figure 1). A dental examination revealed absent teeth, and significant nasal damage was observed. His hair appeared dry and sparse, with numerous healed scars from previous injuries. A comprehensive neurological examination revealed a complete loss of pain and temperature sensation in the peripheral regions, whereas vibration and pressure sensations remained intact. Eye examination indicated reduced corneal sensation, dry eyes, and corneal ulcers, further complicating his clinical picture.

## 3 Differential Diagnosis, Investigations, and Management

**FIGURE 2:**
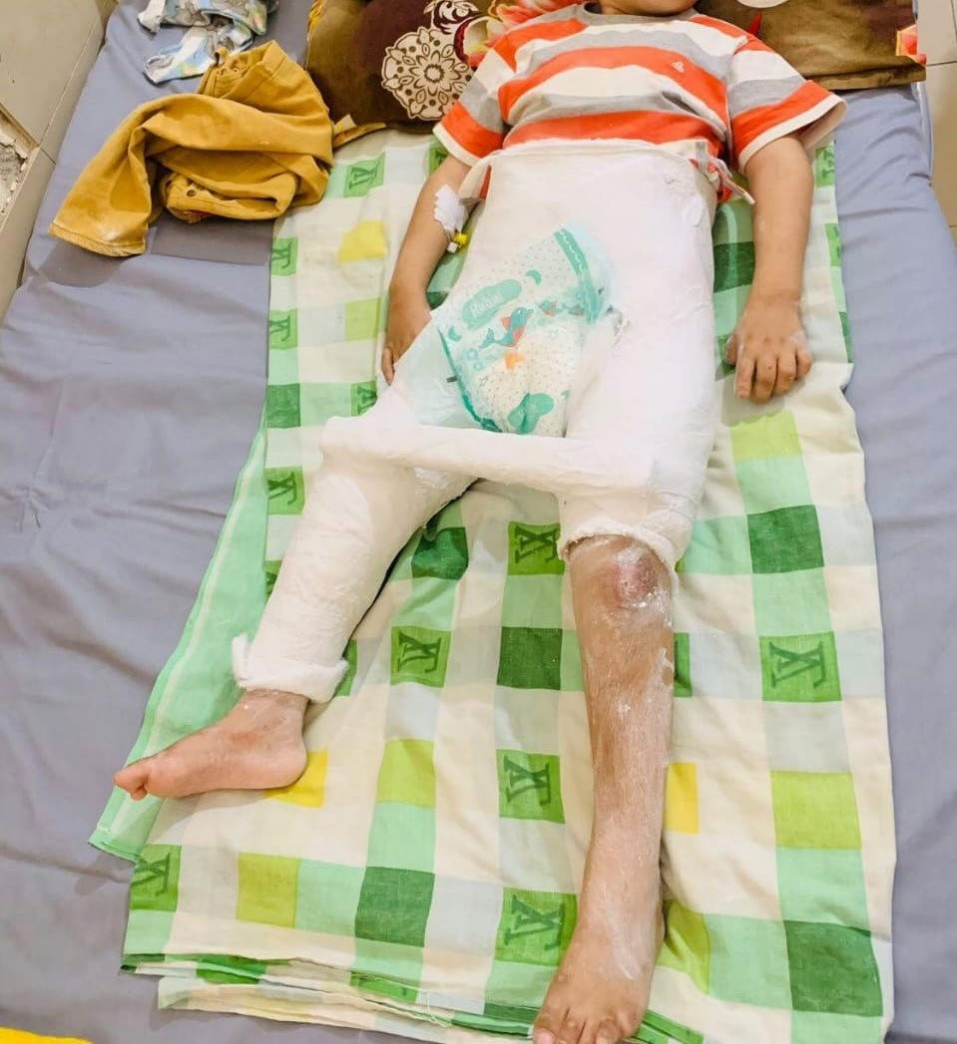
Hip spica cast applied with extra padding for femur fracture.

Laboratory investigations included a complete blood count (CBC), which showed low hemoglobin levels and a high lymphocyte count, raising concerns for potential chronic osteomyelitis. Electrolyte tests indicated borderline sodium levels, whereas potassium and chloride levels were within normal ranges (Table 1). Multiple differential diagnoses that could present in this manner were considered, including Guillain‐Barré syndrome, peripheral nerve injury, leprosy, Fabry disease, Tangier disease, and osteogenesis imperfecta. Familial dysautonomia (HSAN III), characterized by autonomic and sensory dysfunction, and Stuve–Wiedemann syndrome, known for bone deformities and dysautonomia, were also considered because of overlapping features. However, familial dysautonomia was ruled out because the child lacked gastrointestinal symptoms and decreased deep tendon reflexes typical of this condition. Stuve–Wiedemann syndrome was deemed less likely because of the absence of skeletal anomalies commonly associated with the disorder. A major challenge to obtaining a definitive diagnosis was the lack of widespread genetic testing available in Pakistan and its affordability. Nevertheless, genetic testing ultimately confirmed the diagnosis of CIPA, identifying a homozygous mutation in the NTRK1 gene (specific variant: c.851‐33T>A), which is responsible for the development and function of sensory neurons. Given the child's fractured femur, a hip spica cast was applied with extra padding under general anesthesia, as shown in Figure 2. There were no notable adverse events during the procedure.

**TABLE 1:** Relevant lab test values in the patient along with their reference ranges.

Test	Result	Unit	Normal range
Relevant complete blood picture (CBC) parameters			
WBC Count	9.9	10^3^/μL	4.0–11.0
RBC Count	3.28	10^6^/μL	3.50–5.50
Hemoglobin	6.2	g/dL	12.0–16.0
HCT	21.1	%	35.0–50.0
MCV	64.4	fL	76.0–96.0
MCH	18.8	pg	26.0–32.0
MCHC	29.2	g/dL	32.0–36.0
Platelets	216	10^3^/μL	150–450
Relevant differential leukocyte count (DLC) parameters			
Lymphocytes	45.1	%	20.0–40.0
Neutrophils	48.1	%	40.0–80.0
Relevant electrolytes test parameters			
Sodium	146	mEq/L	135–145
Potassium	4.20	mEq/L	3.5–5.0
Chloride	101	mEq/L	95–108

Abbreviations: %, percentage; 10^3^/μL, thousand cells per microliter; 10^6^/μL, million cells per microliter; DLC, differential leukocyte count; fL, femtoliters; g/dL, grams per deciliter; HCT, hematocrit; MCH, mean corpuscular hemoglobin; MCHC, mean corpuscular hemoglobin concentration; MCV, mean corpuscular volume; mEq/L, milliequivalents per liter; pg., picograms; RBC, red blood cell; WBC, white blood cell.

## 4 Outcome and Follow‐Up

The patient was advised to follow‐up in 3 weeks for the removal of the spica cast and an X‐ray to confirm bone healing. The patient was also referred for physiotherapy to improve outcomes and functional recovery. The parents were thoroughly counseled regarding the potential complications associated with CIPA, emphasizing the need for vigilant observation of any unusual behaviors or signs of injury. Regular monitoring of sores and ulcers was advised, especially for ulcerations and erosions of the oral cavity. The parents were educated on the importance of preventing self‐harm and managing the child's body temperature, particularly during fever episodes, to mitigate the risks associated with hyperthermia. They expressed satisfaction with the patient care received and felt better equipped to understand their son's medical condition and prevent further harm.

## 5 Discussion

CIPA is a rare genetic disorder caused by mutations in the NTRK1 gene. This gene encodes the neurotrophic tyrosine kinase receptor type 1, which is essential for the development and survival of sensory and autonomic neurons. Mutations result in the absence of unmyelinated fibers and a significant reduction in small, myelinated fibers, leading to a lack of pain and temperature sensation and autonomic dysfunctions such as anhidrosis [2, 3]. Although mutations in NTRK1 are the primary cause of CIPA, a recent study suggest that variants in other genes, such as SCN9A, may contribute to overlapping phenotypes [4]. This highlights the genetic complexity of CIPA and underscores the importance of comprehensive genetic testing to provide more personalized care for patients.

The presented case of a 5‐year‐old boy with CIPA highlights several characteristic clinical features. The child exhibited profound insensitivity to pain, leading to self‐mutilating behaviors such as biting nails and striking objects, resulting in significant damage to his extremities, including the loss of toes and a thumb. This insensitivity to pain is a hallmark of CIPA and has been extensively documented in the literature [3, 5].

Recurrent unexplained fevers are another common feature of CIPA, as seen in our case. The inability to sweat due to anhidrosis impairs the body's ability to regulate temperature, leading to episodes of hyperpyrexia [1]. Compensatory hyperhidrosis, as observed in our patient, is an adaptive response where sweating occurs in unaffected areas to aid in temperature regulation [6].

Orthopedic complications are frequently reported in patients with CIPA because of repeated trauma and fractures from pain insensitivity. Our patient presented with a fractured femur, a typical injury in these individuals. Chronic osteomyelitis and poor wound healing are common because of repeated injuries and infections [7, 8]. The phenotypic variability in CIPA can lead to differences in the severity of orthopedic complications, as demonstrated in this case.

The patient's medical history included recurrent fevers, insensitivity to pain, and self‐mutilating behaviors beginning in infancy. These behaviors often lead to severe damage, such as the loss of digits and dental complications, as teeth are frequently damaged or lost because of self‐injury. It aligns with existing literature, which reports similar presentations in patients with CIPA, underscoring the severe impact of the disorder on physical health [3, 9]. In addition to physical injuries, patients with CIPA often exhibit intellectual disabilities and developmental delays, affecting the overall quality of life and necessitating comprehensive care [3, 10].

The diagnosis of CIPA is primarily clinical and supported by genetic testing. In the presented case, clinical signs of pain insensitivity, self‐mutilation, recurrent fevers, and anhidrosis raised suspicion for CIPA. Genetic testing confirmed the definitive diagnosis, identifying mutations in the NTRK1 gene. This approach aligns with current diagnostic practices, emphasizing the importance of genetic testing in confirming the diagnosis of rare genetic disorders like CIPA [3, 11].

Management of CIPA is multidisciplinary and focuses on preventing injuries, managing infections, and addressing autonomic dysfunctions. In this case, the fractured femur was managed with a hip spica cast, applied with extra padding to prevent pressure sores. The parents were educated on preventing self‐harm and managing the child's body temperature, particularly during febrile episodes. This approach is crucial as patients with CIPA are at high risk for severe injuries and hyperthermia because of their inability to perceive pain and regulate temperature [3, 12].

Preventive care is essential to minimize the risk of injuries. Protective gear, such as padded clothing and helmets, can help reduce trauma. Regular dental care is also important to manage and prevent dental complications from self‐mutilation. Additionally, vigilant monitoring for signs of infection and prompt treatment is necessary because of the high risk of chronic osteomyelitis [3, 8, 9]. In addition, counseling plays a vital role in managing CIPA. Parents and caregivers must be educated about the nature of the disorder, potential complications, and strategies to prevent injuries. In the presented case, the parents were thoroughly counseled on observing for signs of injury and unusual behaviors, which is critical in preventing severe complications. Genetic counseling is also important for families with a history of CIPA to inform them about the risks of recurrence and the availability of prenatal testing [2].

Research into CIPA is ongoing, with efforts focused on understanding the underlying genetic mechanisms and developing potential treatments. Advances in gene therapy and neuroprotective strategies offer hope for future interventions that could mitigate the effects of NTRK1 mutations. Additionally, early diagnosis through genetic screening can improve outcomes by enabling prompt management and preventive measures [2, 3]. Although our case report offers valuable insights into the clinical presentation, diagnosis, and management of CIPA, the findings may not be applicable to all patients with this condition. Differences in genetic mutations, environmental factors, and access to health care can result in varying manifestations and outcomes in other individuals with CIPA. Hence, there is a need for more advanced research into the topic.

## 6 Conclusion

In summary, CIPA is a rare and debilitating disorder that poses significant challenges in diagnosis and management. The case of the 5‐year‐old boy highlights the critical need for a comprehensive, multidisciplinary approach to care. Early genetic diagnosis, vigilant monitoring, and proactive management of complications are essential in mitigating the impacts of this disorder. Continued research into the genetic and molecular mechanisms of CIPA holds promise for future therapeutic advances, offering hope for better outcomes in affected individuals.

## Author Contributions

**Zubair Amin:** conceptualization, data curation, formal analysis, investigation, project administration, resources, supervision, validation, visualization, writing – original draft. **Humza Saeed:** data curation, formal analysis, investigation, methodology, project administration, supervision, validation, visualization, writing – original draft, writing – review and editing. **Syed Imam Naufil:** project administration, resources, supervision, validation, visualization, writing – original draft. **Sadaf Saba:** formal analysis, project administration, supervision, validation, visualization, writing – original draft. **Abdullah Imtiaz:** conceptualization, supervision, validation, visualization, writing – original draft. **Syed Hassan Akhlaq:** supervision, validation, visualization, writing – original draft, writing – review and editing. **Muhammad Husnain Ahmad:** validation, visualization, writing – original draft, writing – review and editing. **Masab Ali:** formal analysis, validation, visualization, writing – review and editing.

## Consent

Written informed consent was obtained from the patient's parent to publish this report, in accordance with the journal's patient consent policy.

## Conflicts of Interest

The authors declare no conflicts of interest.

## Data Availability

Data and materials are available upon request from the corresponding author.

## References

[1] A. G. Swanson , “Congenital Insensitivity to Pain With Anhydrosis. A Unique Syndrome in Two Male Siblings,” Archives of Neurology 8 (1963): 299–306, 10.1001/archneur.1963.00460030083008. 13979626

[2] Y. Indo , “NTRK1 Congenital Insensitivity to Pain With Anhidrosis,” in GeneReviews®, eds. M. P. Adam , J. Feldman , G. M. Mirzaa , et al. (Seattle: University of Washington, 1993), http://www.ncbi.nlm.nih.gov/books/NBK1769/. 20301726

[3] R. Rodríguez‐Blanque , L. M. Nielsen , B. Piqueras‐Sola , et al., “A Systematic Review of Congenital Insensitivity to Pain, a Rare Disease,” Journal of Personalized Medicine 14, no. 6 (2024): 570, 10.3390/jpm14060570. 38929791 PMC11204641

[4] M. Romagnuolo , C. Moltrasio , R. Cavalli , M. Brena , and G. Tadini , “A Novel Mutation in the Gene Associated With Congenital Insensitivity to Pain, Anhidrosis, and Mild Cognitive Impairment,” Pediatric Dermatology 41, no. 1 (2024): 80–83, 10.1111/pde.15366. 37345838

[5] E. M. Nagasako , A. L. Oaklander , and R. H. Dworkin , “Congenital Insensitivity to Pain: An Update,” Pain 101, no. 3 (2003): 213–219, 10.1016/S0304-3959(02)00482-7. 12583863

[6] A. Rout , S. Arora , S. S. Dalal , and S. Kumar , “Congenital Insensitivity to Pain With Anhidrosis and Compensatory Hyperhidrosis,” Indian Journal of Paediatric Dermatology 22, no. 3 (2021): 260, 10.4103/ijpd.IJPD_107_20.

[7] A. Rotthier , J. Baets , V. Timmerman , and K. Janssens , “Mechanisms of Disease in Hereditary Sensory and Autonomic Neuropathies,” Nature Reviews. Neurology 8, no. 2 (2012): 73–85, 10.1038/nrneurol.2011.227. 22270030

[8] H. Kamil , R. Alassri , D. Albelal , A. B. Alassri , N. Martini , and J. Mahmod , “A Challenging Diagnosis of Chronic Osteomyelitis in a Child With Congenital Insensitivity to Pain: A Case Report,” Annals of Medicine and Surgery 86, no. 5 (2024): 3113–3116, 10.1097/MS9.0000000000001971. 38694364 PMC11060246

[9] A. Safari , A. A. Khaledi , and M. Vojdani , “Congenital Insensitivity to Pain With Anhidrosis (CIPA): A Case Report,” Iranian Red Crescent Medical Journal 13, no. 2 (2011): 134–138. 22737448 PMC3371914

[10] Z. Liu , J. Liu , G. Liu , et al., “Phenotypic Heterogeneity of Intellectual Disability in Patients With Congenital Insensitivity to Pain With Anhidrosis: A Case Report and Literature Review,” Journal of International Medical Research 46, no. 6 (2018): 2445–2457, 10.1177/0300060517747164. 29619836 PMC6023048

[11] K. Daneshjou , H. Jafarieh , and S. R. Raaeskarami , “Congenital Insensitivity to Pain and Anhydrosis (CIPA) Syndrome; A Report of 4 Cases,” Iranian Journal of Pediatrics 22, no. 3 (2012): 412–416. 23400697 PMC3564101

[12] M. Mifsud , M. Spiteri , K. Camilleri , M. Bonello , T. Azzopardi , and M. Abela , “The Orthopedic Manifestations of Congenital Insensitivity to Pain: A Population‐Based Study,” Indian Journal of Orthopaedics 53, no. 5 (2019): 665–673, 10.4103/ortho.IJOrtho_378_18. 31488938 PMC6699213

